# Methods of Minimizing Polycyclic Aromatic Hydrocarbon Content in Homogenized Smoked Meat Sausages Using Different Casings and Variants of Meat-Fat Raw Material

**DOI:** 10.3390/foods12224120

**Published:** 2023-11-14

**Authors:** Marta Ciecierska, Krzysztof Dasiewicz, Rafał Wołosiak

**Affiliations:** Department of Food Technology and Evaluation, Institute of Food Sciences, Warsaw University of Life Sciences, Nowoursynowska 159 Street, 02-787 Warsaw, Poland; krzysztof_dasiewicz@sggw.edu.pl (K.D.); rafal_wolosiak@sggw.edu.pl (R.W.)

**Keywords:** PAHs, smoked meat sausages, casings, raw material, food safety, QuEChERS method, HPLC–FLD/DAD

## Abstract

To ensure food safety and protect human health, the levels of polycyclic aromatic hydrocarbon (PAH) contamination in model smoked-pork meat products were examined to select which type of casing and variant of raw material contributes to minimizing the content of PAHs in the final products. The sausages were smoked in a steam smoke chamber with an external smoke generator. The determination of PAHs was performed using the QuEChERS–HPLC–FLD/DAD method. The analyzed products met the requirements of Commission Regulation (EU) No. 835/2011 on the maximum permissible levels of PAHs. Statistically higher sums of 19 PAHs, including 15 heavy and 4 marker PAHs, were stated in smoked sausages in natural and cellulose casings. Synthetic casings like collagen and polyamide exhibited better barriers against PAH contamination than cellulose and natural casings. For each type of casing, significantly higher concentrations of PAHs were found in the external parts of the products. An increase in the fat content of the raw material increased the levels of PAH contamination in the products, regardless of the casing. Therefore, in industrial practice, the selection of an appropriate type of casing and raw material with the lowest possible fat content can be an effective method for reducing PAH levels in the interior of smoked meat products.

## 1. Introduction

Polycyclic aromatic hydrocarbons (PAHs) represent a diverse class of toxic chemicals ubiquitous in the environment, originating from incomplete combustion or the pyrolysis of organic matter in industrial and human activities [1,2,3,4,5]. The scientific literature consistently confirms the presence of PAHs in various environmental components, consequently leading to their presence in food products [5,6,7,8,9,10,11]. Two primary sources of food contamination by PAHs are environmental deposition and the thermal treatment involved in food processing and preparation for consumption. So far, there is no exact mechanism responsible for PAH formation. However, three potential mechanisms concerning the pyrolysis of hydrocarbons in food, the combustion of cooking fuel, and fat droplets over an open flame are commonly acknowledged as the primary contributors to PAH formation in processed food [3,12,13]. Notably, smoking, grilling, roasting, and direct drying are significant contributors to elevated food contamination levels [14,15,16,17,18].

In December 2002, the European Union Scientific Committee on Food (SCF) identified 15 heavy PAHs as genotoxic carcinogens (SCF PAHs) [14]. In comparison to the light polyarenes from the 16 PAHs on the US EPA list (the United States Environmental Protection Agency), these compounds are much more toxic and stable. Therefore, in February 2005, the European Commission recommended further analysis of these heavy PAHs in foodstuffs [19]. Subsequently, the European Food Safety Authority (EFSA), in its opinion [1] and Commission Regulation (EU) No. 835/2011 [20] concurred that four heavy PAHs, i.e., benzo[a]pyrene, benzo[a]anthracene, benzo[b]fluoranthene, and chrysene, are the best markers for assessing the occurrence of PAHs in food. Consequently, the control and monitoring of these compounds in food are necessary [1,20].

Smoking, an ancient preservation method, imparts a distinctive aroma, taste, and color to meat products through the diffusion of wood combustion-derived volatiles into the meat, usually via natural or artificial casings. However, this process can lead to the formation of polycyclic aromatic hydrocarbons (PAHs) due to the above-mentioned incomplete wood combustion [5,16,18,21,22]. The existing literature on PAH occurrence in smoked meat products offers information about large differentiation in the qualitative and quantitative contamination profile, influenced by many factors [23,24,25,26,27]. Among these factors, we can generally mention smoking conditions, like wood type, its moisture content, combustion temperature, the oxygen level in the smoking chambers, the type of smoke generator, and consequently, the smoking method—direct, where the smoke is generated directly in the smoking chamber (the traditional method), and indirect, where smoking takes place in the chamber with an external smoke generator (the industrial method) [23,27,28,29,30]. Studies have shown that traditionally smoked meat products can contain relatively high, sometimes even alarming levels of PAHs. Additionally, the quality and safety of smoked meats primarily depend on the raw materials. Furthermore, scientific reports indicate that casing types affect both diffusion and deposition of smoke components, including PAHs, in selected types of meat products [31,32,33,34]. 

Due to the high contamination risk in smoked meat products and their significant contribution to consumer PAH intake, maximum permissible levels for PAHs, including the sum of four heavy PAHs and B[a]P, were set in Commission Regulation (EU) No. 835/2011. Additionally, the regulation mandates regular monitoring of PAHs in this group of foodstuffs, indicating a potential for future reductions in the maximum allowable levels, which have been in force since 2014 [20].

Studies investigating the occurrence of PAHs, their formation, as well as treatments and methods aimed at preventing and reducing PAH contamination in smoked meat products, are essential. Although the issues related to minimizing PAH levels in smoked meat products are discussed in various scientific publications, they mainly concern the determination of PAHs from the list of 16 EPA PAHs. However, this list should be considered outdated because, following the opinions of the SCF, EFSA, and Commission Regulation (EU) No. 835/2011, scientists have been recommended to focus on monitoring the much more toxic PAHs on the SCF list [1,14,19,20]. Moreover, data on PAH content and methods for minimizing them in homogenized smoked meat products using different casings and the selection of raw materials are limited. Therefore, the scope of this research was the production of model homogenized pork sausages smoked in a steam smoke chamber with an external smoke generator. Subsequently, PAH determination was carried out using the modern QuEChERS extraction method and liquid chromatography with selective detectors (the QuEChERS–HPLC–FLD/DAD method). The main aim of this work was to select which type of casing and variant of raw material contributed to minimizing PAH content in the final products. In light of the above, PAH determination included 15 SCF PAHs, with 4 marker PAHs, as well as the 4 light PAHs from the EPA list, which are usually predominant in PAH contamination profiles and were additionally analyzed, mainly for comparison purposes with previous works. Overall, the study provides insights into the factors affecting PAH contamination in homogenized smoked sausages and suggests strategies for minimizing health risks associated with PAH consumption.

## 2. Materials and Methods

### 2.1. Research Material and Experimental Design

The materials investigated were homogenized pork sausages, which were produced and smoked in the Division of Meat Technology at the Institute of Food Sciences (WULS, Poland). The meat raw material was purchased at the local market. For the production of the model standardized pork batter, the following ingredients were used: cooled (4 ± 1 °C) pork ham (quadriceps muscles) of class I and pork throat without skin in a ratio of 70:30 of the meat-to-fat raw material, respectively (variant 1). Additionally, water/ice (35%), a curing mixture (99.4% of NaCl and 0.6% of NaNO_2_; in the amount of 2.2%), soy protein (1.5%; AMCO Sp. z o.o., Dybów-Kolonia, Poland), spices (a mixture of black pepper and herbal pepper in the ratio 1:2, in the amount of 0.6%; Kamis, Wólka Kosowska, Poland), polyphosphate preparation (0.52%; Tari P31; BK Giulini, Ladenburg, Germany), and sodium isoascorbate (0.05%) were added to the meat batter weight. The meat batter prepared in this way consists of the basis for the production of experimental model homogenized smoked-pork sausages.

The scope of the technological production of sausages included the preparation of four types of sausages with the same composition (from the standardized pork batter of variant 1) but differing in the casing used. The meat batter was stuffed in four different casings: natural (sheep intestine), cellulose, collagen, and polyamide, all 19–22 mm in diameter (PROMAR PPH Sp. z o.o., Zawiercie, Poland). The casings used had been designed for use in smoked products. Additionally, control variants of the sausages in two selected types of casings—natural and artificial (polyamide), and not subjected to the smoking process—were prepared. Therefore, for this purpose, the casings with potentially the lowest and the highest degree of barrier against oxygen, air, smoke, and water vapor, as indicated by their manufacturer, were used.

Apart from the standardized pork batter of variant 1 (70:30 of meat-to-fat raw material), two other variants were produced in natural and cellulose casings because in analyzed smoked sausages of variant 1, they led to the highest and the lowest levels of total PAH contamination. These were variant 2 and variant 3 with 60:40 and 50:50 of meat-to-fat raw material, respectively.

To study the diffusion of PAHs into the sausage interior as a result of the smoking process, their content on the surface (the external part with the skin) and in the internal part of the product (the remaining part after removing the external part with the skin) was analyzed. 

### 2.2. Production Technology of Model Homogenized Smoked-Pork Sausages

**Figure 1:**
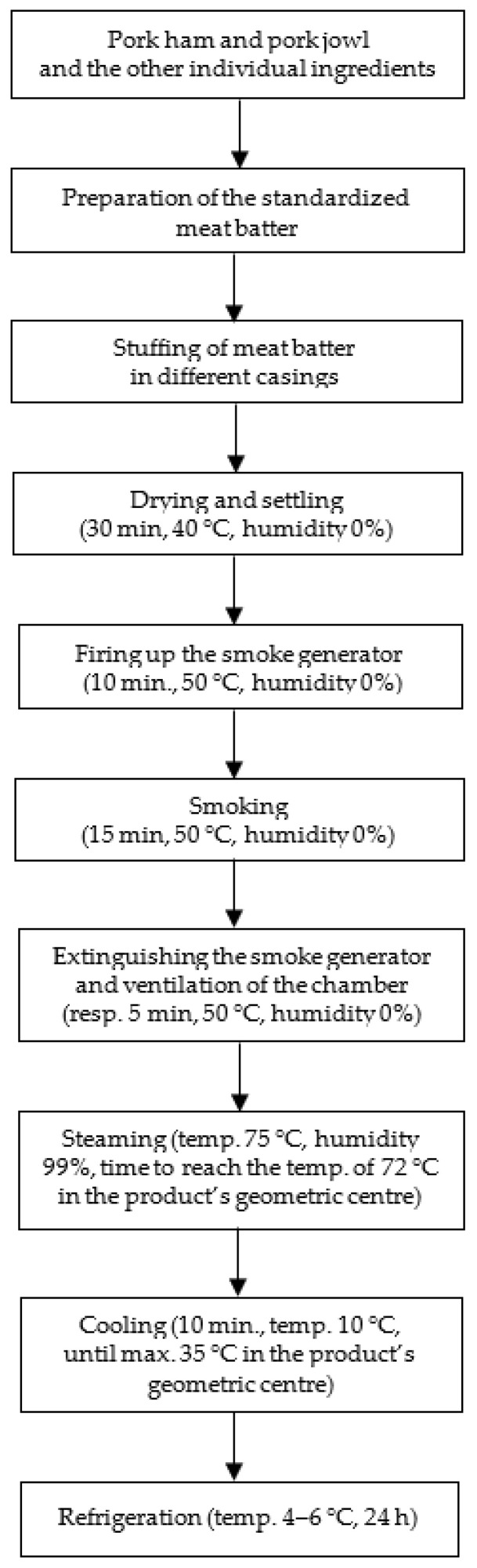
Scheme of production technology of model homogenized smoked-pork sausages.

**Figure 2:**
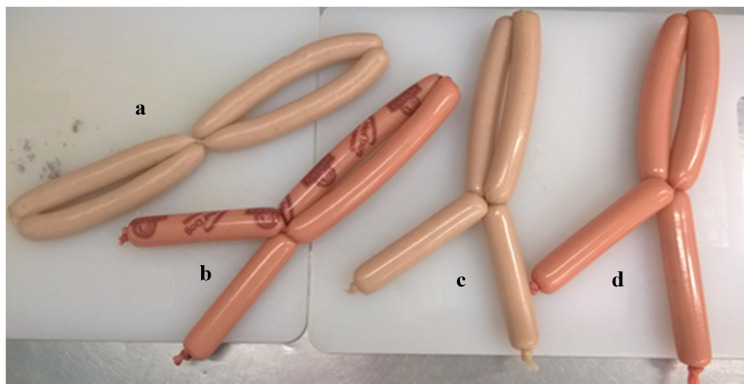
Model homogenized pork sausages in various casings ((**a**) natural, (**b**) cellulose, (**c**) collagen, (**d**) polyamide) before the smoking process.

The production of sausages was carried out in three batches following a constant production scheme, which is presented in Figure 1. Initially, the meat and fat raw materials were cut and ground using a Mesko WN60 laboratory grinder (Mesko AL. 2–4, MESKO-AGD Sp. z o.o., Skarżysko-Kamienna, Poland) through a mesh with a hole diameter of 3 mm Subsequently, all ingredients were weighed according to the above recipe. The next stage was performed under vacuum conditions in a cutter, Stephan UM5 Universal Machine (A. Stephan u. Sohne GmbH & Co., Hameln, Germany), at a constant speed of 3000 rpm. During this stage, the ingredients were added and mixed in the following order. First, pork quadriceps muscles were mixed with polyphosphate preparation. The mixing time was about 10–15 s. In the next step, ¾ portion of ice, curing mixture, soy protein, and spices were added and mixed for about 1 min. In the third phase, sodium isoascorbate, pork throat, and ¼ portion of ice were added and mixed for approx. 1 min. The final temperature of the meat batter did not exceed 12 °C. Following this, the casings were filled with the prepared meat batter using an F. Dick Sausage Stuffer (F. Dick GmbH, Deizisau, Germany), resulting in sausage bars with an average length of 10 to 15 cm (Figure 2).

The sausages were smoked in a steam smoke chamber equipped with an external smoke generator of JUGEMA (Środa Wielkopolska, Poland) using oak chips at a standard smoke density. The stages of thermal treatment included drying and settling (30 min, temp. 40 °C, humidity 0%), firing up the smoke generator (10 min, temp. 50 °C, humidity 0%), smoking (15 min, temp. 50 °C, humidity 0%), extinguishing the smoke generator (5 min, temp. 50 °C, humidity 0%), ventilation of the chamber—smoke evacuation using specialized filters (5 min, temp. 50 °C, humidity 0%), steaming (time necessary to reach the temperature of 72 °C in the geometric center of the product, temp. 75 °C, humidity 99%), cooling (10 min, temp. 10 °C, until the geometric center of the product reached the temperature no higher than 35 °C), followed by transfer to a cold store, and further cooling (temp. 4–6 °C, 24 h). 

Each sample from three production batches was analyzed in four repetitions.

### 2.3. Chemicals and Materials

Acetonitrile (HPLC gradient grade), anhydrous magnesium sulfate, and sodium chloride (both of analytical purity ˃ 99.0%) were purchased from Avantor Performance Materials Poland S.A. (Gliwice, Poland). Sorbents for the QuEChERS method: Sepra PSA Bulk Packing (primary–secondary amine) and Sepra C18-E Bulk Packing (silica gel modified with C18 groups) were provided by Phenomenex (Warsaw, Poland). Deionized water was obtained from a Millipore Milli-Q water purification system. Polytetrafluoroethylene (PTFE) syringe filters (25 mm i.d., 1 µm pore size) and centrifuge (PTFE) tubes were provided by Bio Analytic (Gdańsk, Poland).

A standard mix of 15 PAHs from the SCF list (PAH-Mix 183, Dr Ehrenstorfer) and 16 PAHs from the US EPA list (PAH-Mix 9, Dr Ehrenstorfer) was supplied by Witko (Łódź, Poland). 15 SCF PAH mixture included the following compounds: cyclopenta[c,d]pyrene (C[cd]P), benzo[a]anthracene (B[a]A), chrysene (Chr), 5-metylchrysene (5-MChr), benzo[j]fluoranthene (B[j]F), benzo[b]fluoranthene (B[b]F), benzo[k]fluoranthene (B[k]F), benzo[a]pyrene (B[a]P), dibenzo[a,h]anthracene (D[ah]A), benzo[g,h,i]perylene (B[ghi]P), indeno[c,d]pyrene (I[cd]P), dibenzo[a,l]pyrene (D[al]P), dibenzo[a,e]pyrene (D[ae]P), dibenzo[a,i]pyrene (D[ai]P), and dibenzo[a,h]pyrene (D[ah]P). The second standard mix was used for the analysis of 4 light PAHs—phenanthrene (Phen), anthracene (Anthr), fluoranthene (F), and pyrene (Pyr).

### 2.4. Thermal Efficiency of the Smoking Process of Model Homogenized Smoked-Pork Sausages

After the smoking process, the values of the thermal efficiency of the final products of variant 1 in different casings, derived from three smoking batches, were calculated. This calculation involved determining the ratio of the weight of the cooled smoked sausage to its weight before the smoking process, expressed as a percentage.

### 2.5. Determination of PAHs with the QuEChERS–HPLC–FLD/DAD Method

The preparation of samples for the qualitative and quantitative analysis of PAHs was based on the methodology of Shelly and Perman [35] with some modifications. These included extraction of PAHs and purification of the sample using the QuEChERS method and chromatographic analysis utilizing the HPLC–FLD/DAD technique.

To extract fat and PAHs, approximately 5 g of homogenized smoked sausage sample was weighed into a 50 mL centrifuge tube. It was then poured with 10 mL of acetonitrile and intensively mixed for 1 min on a vortex. Following this, 1 g of sodium chloride and 4 g of magnesium sulfate were added to the tube. The sample was subjected to vortex mixing for 3 min and then centrifuged in an MPW-251 laboratory centrifuge (Warsaw, Poland) for 3 min at 3400 rpm. After this process, 4 mL of the obtained extract, aimed at purification and isolation of PAH fraction, was transferred to a 15 mL centrifuge tube containing previously added sorbents: 900 mg MgSO_4_, 300 mg PSA, and 150 mg C18. The tube content was mixed on a vortex for 3 min and then centrifuged for 3 min in the laboratory centrifuge mentioned above at 3400 rpm. Subsequently, the obtained supernatant was filtered through the PTFE filter (pore diameter 0.20 μm) to a chromatography vial and subjected to chromatographic analysis.

The determination of PAHs was conducted based on the method described by Ciecierska [10] with some modifications, using Shimadzu HPLC (Kyoto, Japan), consisting of liquid chromatography LC-10ATVP, degasser DGU-14A, auto-injector SIL-10ADVP, fluorescence detector RF-10AXL, diode array detector SPD-M10AVP and system controller SCL-10AVP. Data collection and analysis were conducted using the LabSolution 2.1 program. PAH separation was performed on a Restek Pinnacle II PAH column (150 × 3.2 mm, 4 µm, Anchem Plus, Warsaw, Poland) at 30 °C with a gradient elution of acetonitrile/water (70:30, *v*/*v*; A) and acetonitrile (B). The flow rate was 1.5 mL/min. The following gradient elution program was applied: 0–3 min 0% B to 10% B, 3–10 min 10% B to 100% B, 10–24.0 min 100% B, and 24–27 min 100% B to 0% B. Different excitation and emission wavelengths for PAH fluorescence detection were used: 256/370 nm (Phen, Anthr), 270/420 nm (F, Pyr, B[a]A, Chr, 5-MChr, B[b]F, B[k]F, B[a]P, D[ah]A, D[al]P, B[ghi]P, D[ae]P), 270/500 nm (B[j]F, I[cd]P), and 270/470 nm (D[ai]P, D[ah]P). To detect C[cd]P, 254 nm of wavelength at the diode array detection was applied.

### 2.6. Quantification and Validation of QuEChERS–HPLC–FLD/DAD Method 

Qualitative and quantitative analysis of PAHs using an external standard method and validation of the QuEChERS–HPLC–FLD/DAD method was conducted based on the procedure published by Ciecierska [10]. Two previously described PAH standard mixtures (PAH-Mix 183, PAH-Mix 9, Dr Ehrenstorfer) were applied. Six PAH standard solutions at different concentration levels (1–50 µg/L) were prepared to establish the calibration curves for particular PAHs. The method linearity in this concentration range was confirmed for most PAHs, whereas, in the range of 2–50 µg/L, it was only for a few compounds (Table 1). Validation parameters, including the limit of detection (LOD), the limit of quantification (LOQ), recovery values with relative standard deviation (RSD), and HORRAT_R_ values (a measure of method precision) for 19 analyzed PAHs, were calculated in accordance with Commission Regulation (EU) No. 836/2011 [36], and are presented in Table 1. For recovery experiments, one of the smoked meat model samples (variant 1, internal part of sausage in a natural casing) was spiked with three different levels of PAH standard mixtures (1, 10, and 100 µg/kg). Both the fortified sausages and unfortified ones were analyzed thrice. The method validation parameters for PAH determination in the smoked meat model sample are shown in Table 1.

**Figure 3:**
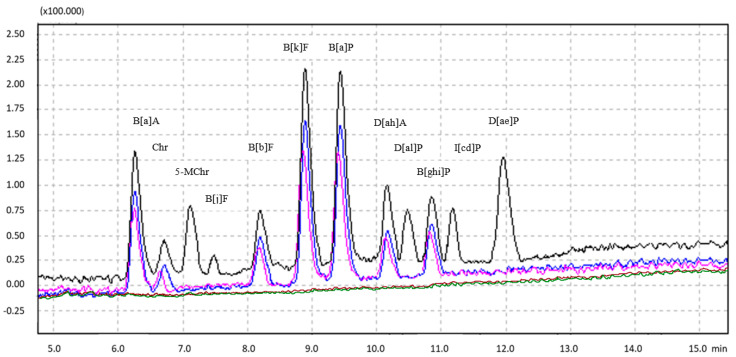
The HPLC−FLD chromatograms of PAHs listed by SCF (PAH−Mix 183, Dr Ehrenstorfer, 5 pg/µL; black color) and external parts of smoked homogenized pork sausages of variant 1 in a natural casing (blue), cellulose casing (pink), collagen casing (brown) and polyamide casing (green).

All obtained validation parameters, including LOD, LOQ, recovery, and HORRAT_R_, proved that the QuEChERS–HPLC–FLD/DAD method meets the requirements of Commission Regulation (EU) No. 836/2011 [36] concerning the methods of 4 marker PAH analysis in foodstuffs. Furthermore, satisfactory validation parameters were also stated for the analyzed compounds from the SCF list and the 4 light PAHs from the EPA list (Table 1). The chromatograms of the analyzed PAHs listed by the SCF and external parts of smoked homogenized pork sausages of variant 1 in natural, cellulose, collagen, and polyamide casing are presented in Figure 3.

### 2.7. Statistical Analysis

The obtained results were subjected to statistical analysis using Statistica ver. 10 PL (StatSoft, Inc., Tulsa, OK, USA). Multiple comparison analysis with Tukey’s test, at a significance level α = 0.05, was used to assess the significance of the differences in the mean PAH contents among analyzed samples with respect to different casings, parts of the products, and product variants.

## 3. Results

### 3.1. Analysis of the Thermal Efficiency of the Smoking Process of Model Homogenized Smoked-Pork Sausages

Due to the smoking process, the weight of the analyzed products decreased. The mean values of thermal efficiency were determined within a range of 81.1 to 84.5%, depending on the type of casing used (Table 2). The highest weight loss, equal to 18.9%, and simultaneously, the lowest thermal efficiency, at a level of 81.1%, were found in the sausages in natural casings. This type of casing is characterized by a low barrier to both smoke and water vapor [37], attributed to its high water vapor transmission rate (1800 g/m^2^) and gas transmission rate (750 cm^3^/m^2^). This makes it widely used in meat processing, especially during smoking and drying [38]. However, the substantial diffusion associated with natural casings may contribute to the lower thermal efficiency of the smoking process. The lowest weight loss (15.5%) and simultaneously the highest thermal efficiency (84.5%) were obtained in smoked sausages in an artificial polyamide casing. This aligns with the high barrier properties of polyamide casings against water vapor. The sausages in cellulose and collagen casings exhibited thermal efficiencies of 82.5 and 82.7%, respectively.

The average thermal efficiencies of the three smoking batches for each batch of the product were similar, affirming the repeatability of the process (Table 2). Thermal efficiency was also determined for the control variant of sausages in natural casing, which was not subjected to the smoking process and was equal to 91.1%. This confirms a higher loss of product weight after smoking. 

It was stated that the transmission rates of both water vapor and smoke, as well as light components, affect the weight loss and, consequently, the production efficiency of the product [39]. It also leads to changes in the chemical composition, especially in fats, or changes in water activity, significantly influencing not only the quality but also the safety of final products. The increase in mass losses during a steaming process after smoking is directly correlated with the rise in temperature, as previously smoked product loses more water. Sobczak et al. [40] confirmed a significant decrease in product weight, approximately 14–24% during the final production stage. Dolatowski and Skórnicki [41], in their study of smoked sausages, noted that the weight loss of the product can even reach 30%. In light of the above, the technological process carried out in this study can be considered efficient and effective. The obtained average production efficiency is comparable to the results published in other literature sources.

### 3.2. Analysis of PAH Contamination of Model Homogenized Smoked-Pork Sausages

Results of the mean content of PAHs in model smoked homogenized pork sausages of variant 1, including the type of casing and the part of the product, are presented in Table 3. The concentrations of PAHs in the sausages of the other two product variants in the two selected types of casings, also presenting contents for particular parts of the product, are shown in Table 4 and Table 5. Data for individual compounds and the sum of all analyzed PAHs, including the sum of 15 heavy PAHs, 4 light PAHs from the EPA list, and 4 heavy and marker SCF PAHs, are given.

The obtained results confirmed the lack of statistically significant differences in the content of analyzed PAHs, including the sums of 15 heavy PAHs, 4 light, and 4 marker-heavy PAHs, as well as B[a]P, between three smoking batches. It proves the repeatability of the smoking process and the precision of the measurement method used. Therefore, the presented results are the mean contents derived from 12 samples of each product type, analyzed from three production batches and four repetitions. Additionally, PAH analysis of the control variants of sausages of variant 1 in two selected types of casings (natural and polyamide) and not subjected to the smoking process revealed no contamination with heavy PAHs and only trace amounts of light polyarenes.

Examining the qualitative profiles of polyarene content in the model smoked-pork sausages of variant 1, it can be noted that only for external parts of products in natural and cellulose casings, apart from the predominant 4 light PAHs, some of the 15 heavy PAHs and simultaneously 4 marker PAHs were detected (Table 3). Among them, B[a]A, Chr, B[b]F, B[k]F, B[a]P, D[ah]A, and B[ghi]P were found, as depicted in the chromatograms in Figure 3. The 4 light PAHs constituted 79% and 80% of all investigated PAHs, whereas 15 heavy PAHs averaged 21% and 20% of the total content of PAHs, respectively, for external parts of sausages in natural and cellulose casings. The 4 marker-heavy PAHs constituted 12% of the total 19 PAHs content in these samples. In the sausages stuffed in collagen and polyamide casings, heavy PAHs were not detected (Figure 3), but they only contained 4 light PAHs. However, in the chromatograms, light PAHs were intentionally not shown, whereas only heavy PAHs were presented. Otherwise, the dominant character of light PAHs in the qualitative-quantitative profiles would mean that there would be no visible differences between the content of heavy PAHs in the analyzed samples. Therefore, it was desirable to show and highlight in the chromatograms the differences between them in terms of only marker-heavy PAHs, one of the most toxic polyarenes (Figure 3). Qualitative profiles of PAHs were also similar in internal parts of all sausages, regardless of the casing used, and even in external parts of the products stuffed in collagen and polyamide casings since only 4 light PAHs were detected. Analyzing the qualitative profiles of PAHs in two other sausage variants, characterized by a higher fat content compared to variant 1 and stuffed in two selected types of casings, it can be stated that they are similar to each other (Table 4 and Table 5). The percentage of 4 light PAHs and 15 heavy PAHs was in a range of 78–80% and 20–22%, respectively, for external parts of the sausages of variant 2 and 3 in natural and cellulose casings. The sum of 4 heavy and marker PAHs averaged from 11 to 13% of the total content of PAHs in these samples. Furthermore, no heavy PAHs were detected in the inner parts of any of the sausage variants in the cellulose casing. Only for the natural casing in the internal part of variant 3 was the presence of the same heavy PAHs, like B[a]A, Chr, B[b]F, B[k]F, B[a]P, D[ah]A and B[ghi]P, as in the outer part, noted, but at very low levels. The percentage of 15 heavy and 4 light PAHs constituted 5% and 95%, respectively, of all polyarenes under investigation.

The type of casing used in the production of sausages from the standardized pork batter of variant 1 (70:30 of meat-to-fat raw material) contributed to the highest level of contamination. It should be noted that among the analyzed smoked sausages, statistically, the most contaminated with the sum of 19 PAHs were the sausages in the natural casing—both their external and internal parts. These levels were equal to, respectively, 82.68 and 18.75 μg/kg. Also relatively highly contaminated but statistically lower were the sausages in cellulose casing. The total mean 19 PAH concentrations were determined at a level of 63.14 and 10.06 μg/kg for their exteriors and interiors. Smoked products in polyamide and collagen casings were statistically the least contaminated. The levels of total PAH contamination, being equal to 14.99 and 1.63 μg/kg and 13.52 and 1.46 μg/kg, were found in external and internal parts of the sausages, respectively, in polyamide and collagen casings. Therefore, statistically significant differences were proved between the analyzed sausages stuffed in different casings.

Regarding the total 15 PAH contamination in the smoked sausages of variant 1, it was noted that natural casing also led to the highest level of contamination (16.99 μg/kg). It concerned only their exteriors, as no heavy PAHs were detected in the internal parts for all casings. Statistically less contaminated with 15 heavy PAHs were the sausages in the cellulose casing (12.74 μg/kg). In the case of polyamide and collagen casings, the sausage exteriors were not contaminated by heavy PAHs.

When analyzing smoked sausages of variant 1 in various casings, statistically significant differences were found in the levels of 4 marker-heavy PAHs. Similar to the 15 PAHs, these four compounds contaminated only the external parts of sausages stuffed in natural and cellulose casings. The mean sums of 4 heavy PAH contents were, respectively, 10.10 and 7.39 μg/kg. Additionally, concerning the mean B[a]P contents, the exteriors of sausages in natural casing were statistically more contaminated (2.34 μg/kg) compared to cellulose casing (1.79 μg/kg). The levels of contamination of the model smoked sausages by the sum of 4 heavy and marker PAHs did not exceed the maximum permissible level set in Commission Regulation (EU) No. 835/2011 [20], which is 12 μg/kg of the product. Analyzing B[a]P contents, despite exceeding the maximum legal limit (2 μg/kg) in the outer part of sausages in a natural casing, the analyzed products can be considered safe for consumption as B[a]P was not detected in their inner parts, regardless of the casing used. 

Comparing the contamination levels of sausages from variant 1 with those produced as two other product variants, variant 2 and variant 3, with, respectively, 60:40 and 50:50 meat-to-fat raw material ratios, stuffed in two selected casings, it can be concluded that the increase in the percentage of fat raw material in meat batter statistically increased the PAHs contamination levels of the smoked products, irrespective of the casing used and the analyzed part of the product (Table 3, Table 4 and Table 5). For the external parts, in the case of variant 1, variant 2, and variant 3, statistically significant differences were confirmed for all four comparisons of the total content of 19 PAHs, 15 heavy PAHs, 4 light, and 4 marker-heavy PAHs, both with natural and cellulose casings. For instance, variant 3 showed statistically the highest levels of 19 PAH contamination (116.32 and 89.95 μg/kg, respectively, for the natural and cellulose casings), variant 2 statistically lower (98.37 and 76.43 μg/kg), and variant 1 the lowest (82.68 and 63.14 μg/kg). Similarly, in the case of the internal parts of the analyzed smoked meat products, variant 3, with the highest percentage of fat in the meat-stuffing recipe, turned out to be the most contaminated with PAHs, whereas variant 1 showed the least contamination.

In the case of both variants with increased fat content in the meat-stuffing recipe, the B[a]P content in the outer part of the tested smoked products, with each of the casings used, exceeded the legal limit of 2 µg/kg of the product (Table 4 and Table 5). Despite exceeding the maximum allowable limit in the sausage exteriors of variants 2 and 3, the internal parts of the products can be considered safe. B[a]P was detected only in the interior of the last variant in natural casing, but at a very low level, equal to 0.24 μg/kg. Regarding the sum of 4 marker-heavy PAHs, only in the sausage exteriors of variant 3 stuffed in natural casing did it exceed the legal limit of 12 µg/kg and was equal to 14.59 µg/kg. However, considering that such final products are consumed whole, it can be concluded that the contamination of model sausages, even from variants with increased fat content in the meat-stuffing recipe, does not pose a health problem according to Commission Regulation (EU) No. 835/2011 [20].

Regarding the diffusion of PAHs into sausage interiors due to the smoking process, based on the statistical analysis, for each product variant, regardless of the casing used, significant differentiation in the sum of 19 PAHs, the sum of 4 light, and the sum of 4 marker-heavy PAHs between external and internal parts of sausages were proven (Table 3, Table 4 and Table 5). Therefore, for every casing in each product variant, statistically higher levels of total 19 PAHs, 4 light PAHs, or 4 heavy PAHs contamination were observed in the product’s exterior. For instance, in the case of variant 1, the sums of 19 PAH content were determined in the external and internal parts of the sausages, respectively, at levels of 82.68 and 18.75 µg/kg in the case of natural casing, 63.14 and 10.06 µg/kg in cellulose casing, 14.99 and 1.63 µg/kg in polyamide casing, and 13.52 and 1.46 µg/kg in collagen casing. Hence, the difference in the sum of 19 PAHs between the exterior and interior of sausages was about 4-fold with natural casing used, 6-fold with cellulose casing, and about 9-fold for both polyamide or collagen casing. In the case of sums of 4 marker-heavy PAHs, the differences in levels between external and internal parts of the tested products were even higher. For only one kind of sample, i.e., the sausage of variant 3 stuffed in the natural casing, apart from the external part, 4 marker PAHs were also found in the internal part of the product, and the level of difference between them was about 19-fold. The type of casing used, as well as the variant of the product, and, more precisely, the fat content in the meat batter, contributed to such a large differentiation in the levels of PAH contamination of particular parts of the smoked sausages. The above-mentioned results confirm the low degree of diffusion of heavy PAHs into the sausage stuffed in natural casing. Moreover, they proved that artificial casings, polyamide, and collagen were characterized by greater barrier properties than cellulose and natural casings.

## 4. Discussion

In summarizing the main objective of this study and identifying the casings with the most significant impact on reducing PAHs, it was confirmed that each type of casing provided a certain degree of protection, and casings made of natural intestine reduced the penetration of hydrocarbons into the product least of all. Škaljac et al. [34] determined the content of PAHs in smoked sausages of two variants, in natural casing (pork intestine) and collagen casing. The researchers analyzed PAHs from the US EPA list. In all samples, the light PAHs averaged 99% of the determined compounds. The highest content of these compounds was found in sausages stuffed in natural casings and smoked in traditional conditions. The lowest content of light PAHs was obtained in sausages smoked in collagen casings under industrial conditions. The investigators found that the total concentration of 13 light PAHs, according to the US EPA list, was significantly higher in sausages stuffed in natural casings (220 μg/kg) compared to sausages in collagen casings (31.3 μg/kg) under the same technological conditions. In other studies on frankfurter-type smoked sausages, the influence of three types of casings on the content of 4 heavy PAHs in the final products was analyzed [25]. Likewise, the levels of 4 heavy PAHs in smoked sausages in cellulose and collagen casings were statistically significantly lower compared to sausages in natural casings. Similar results were also obtained in other studies [33,42], indicating that a high percentage of PAHs remains in the removable, inedible casing and does not penetrate the meat product [25].

The cellulose-based casing used in this study was single-layered, likely contributing to the heightened deposition of PAHs on its surface compared to other artificial casings. Collagen and polyamide casings were a complete barrier against the heavy PAHs. Gomes et al., analyzing traditional dry fermented sausages, stated that safer products with significantly lower PAH contamination levels were obtained when collagen casing was used [31]. Ledesma et al. [32] also found that the different effects of casings on minimizing the PAH content in final products are attributed to differences in their physicochemical properties. The study revealed that the high porosity (approx. 66.8%) of natural casings influences fat penetration from the inside to their external surface. As a result, the casing becomes sticky, moist, and wrinkled. It can be concluded that this effect promotes the adsorption of smoke particles, potentially resulting in increased PAH formation. Smoke particles may also damage the casing and start migrating into the product. However, the low porosity (approx. 16.6%) of synthetic coatings keeps the fat content in the smoked product unchanged. Consequently, the surface of the casing remains dry, non-sticky, and smooth, exhibiting less affinity for smoke particles and, therefore, also for PAH particles. Moreover, the much smaller pore size in artificial casings prevents larger smoke particles from penetrating the product. The pore diameter in the natural animal intestine averages 600 nm, while in collagen casing, it is approximately 48 nm. This discrepancy elucidates the differential penetration of PAHs through natural casings and collagen ones.

Since PAHs always accumulate in fat, the results obtained in this study confirmed that the high-fat products are the most contaminated. This observation supports the higher levels of PAHs in both variants of model sausages with increased fat content in the meat-stuffing recipe. Other researchers also emphasized that among meat products, the fattiest ones are the most contaminated with PAHs and are considered their main source. Other studies proved that lipid compounds are crucial precursors for PAH formation. Consequently, the higher the fat content in raw products, the more PAHs are produced during thermal processing [5,8,13,15,43].

According to Šimko [22], the highest PAH concentrations are found in the outer part of the meat immediately after the smoking process. However, PAHs penetrate the inner part of the product during processing and storage, stabilizing their concentration over time. Lower concentrations of both benzo[a]pyrene and total PAH in the central part of smoked meat products compared to the external part have also been observed in other studies [23,24,25]. Researchers emphasized that this relationship results from the adsorption of smoke particles on the external part, known as surface dryness, leading to reduced penetration of these compounds inside the products. This is also consistent with the findings of this study. Chen et al. [44] found that the ability of PAHs to penetrate the product is negatively correlated with molecular weight. This thesis is also supported by the results of this study, as light PAHs were highly predominant in all analyzed samples, especially in the internal parts, in which none of the PAHs with the highest molecular weight were detected (e.g., indeno[c,d]pyrene, dibenzo[a,l]pyrene, dibenzo[a,e]pyrene, dibenzo[a,e]pyrene, dibenzo[a,i]pyrene, and dibenzo[a,h]pyrene).

Depending on the method, presently commonly performed industrially, either in chambers with an external smoke generator or traditionally with smoke generated directly in the smoking chamber and the conditions of thermal treatment, the levels of B[a]P and other PAHs content vary greatly in smoked meat products [12,15,21,23,24,27,28,30,45]. Apart from the technological conditions of smoking, an important parameter that can differentiate PAH levels is the diameter of the food product. Migdał et al. [46] found that the larger the surface area of a smoked product at a low weight, the higher the PAH content. In the case of benzo[a]pyrene, the level of concentration was up to 9.1 μg/kg, and the sum of 4 PAHs even 70 μg/kg product.

In summary, one of the main reasons for the differences in PAH contamination levels among meat products is the divergence in the technological process, smoking conditions, and the type of wood used [5,28]. This study confirmed that the type of casing contributes to minimizing polyarene levels in the internal part of smoked products. As a result of the mild conditions of the smoking process and three types of artificial casings used—cellulose, collagen, and polyamide—a complete barrier against the penetration of PAHs into the inside of the smoked product was proven. The exception was observed in the case of the fattiest variant of sausage in natural casing, where very low contents of B[a]P, the sum of 4 heavy PAHs, and 15 PAHs were found in the inner part of the product. The increase in the percentage of fat in the meat batter led to higher PAH contamination levels. Therefore, it can be assumed that the use of industrial casings, along with smoking with an external smoke generator, while controlling and potentially reducing fat content in the meat fat raw material significantly affects the safety of smoked meat products, which is an effective strategy for minimizing PAH contamination.

## 5. Conclusions

In conclusion, our study provides valuable insights into the factors influencing PAH contamination in homogenized smoked meat sausages, offering practical implications for producers and consumers to enhance product safety and quality. In addition to using the mildest and safest possible smoking conditions in a steam smoke chamber with an external smoke generator, key strategies for minimizing PAHs in smoked meat products involve appropriate casings, managing fat content in the meat batter, and recognizing the generally safer nature of the inner parts of sausages for consumption, even if levels of PAHs sometimes exceed legal limits in the exterior. Collagen and polyamide casings can act as effective barriers preventing PAH contamination in smoked sausages. The use of artificial casings is recommended to reduce human intake of PAHs through smoked meat products effectively. Additionally, the higher the fat ratio in sausages, the higher the concentrations of PAHs, emphasizing the importance of controlling fat content during food production and preparation. Therefore, consumers can use appropriate ingredients as reference standards when selecting foods, and make conscious decisions to limit exposure to PAHs. Furthermore, since smoked meat products are often consumed and considered the primary source of PAHs, further scientific research on PAH contamination levels and methods of their reduction is still needed. Undoubtedly, they can contribute to improved food safety standards and consumer satisfaction.

## Figures and Tables

**Table 1:** The QuEChERS–HPLC–FLD/DAD method validation parameters for PAH determination in smoked meat model sample.

PAH	Calibration Curve	Correlation Coefficient r^2^	Linearity Range (µg/L)	LOD (µg/kg)	LOQ (µg/kg)	Recovery for 100 µg/kg of Sample Fortification	Recovery for 10 µg/kg of Sample Fortification	Recovery for 1 µg/kg of Sample Fortification	Recovery (%) *	RSD (%) *	HORRAT_R_ Value *
Phen	y = 265,879x + 45,520	0.9999	1–50	0.06	0.11	80.6	78.1	76.4	78.4	8.3	0.7
Anthr	y = 195,739x + 43,120	0.9999	1–50	0.07	0.14	81.5	77.6	74.3	77.8	8.1	0.7
F	y = 29,531x − 2478.5	0.9998	1–50	0.13	0.26	89.0	87.2	81.4	85.9	9.2	0.8
Pyr	y = 128,631x + 55,798.5	0.9998	1–50	0.08	0.16	92.3	89.7	85.6	89.2	9.9	0.8
C[cd]P	y = 221,125x + 11,330	0.9994	2–50	0.47	0.94	109.3	106.9	108.9	108.4	9.1	0.8
B[a]A	y = 268,136x − 14,620.7	0.9997	1–50	0.05	0.10	93.2	87.3	85.9	88.8	6.3	0.5
Chr	y = 72,306x + 5232.1	0.9997	1–50	0.08	0.16	88.4	84.5	85.7	86.2	6.6	0.5
5-MChr	y = 152,197x + 5685.2	0.9996	1–50	0.07	0.15	91.4	84.8	80.6	85.6	7.3	0.6
B[j]F	y = 156,650x + 2996.8	0.9995	2–50	0.32	0.64	83.9	82.1	78.0	81.3	6.8	0.6
B[b]F	y = 119,531x + 55,798.5	0.9996	1–50	0.10	0.20	92.2	87.8	81.7	87.3	7.7	0.6
B[k]F	y = 422,930x + 18,992.4	0.9998	1–50	0.10	0.19	93.1	88.1	84.7	88.7	7.0	0.6
B[a]P	y = 419,310x − 2966.6	0.9998	1–50	0.12	0.24	92.2	90.2	86.1	89.5	7.6	0.6
D[ah]A	y = 122,346x + 3706.9	0.9996	1–50	0.13	0.26	86.0	80.3	78.9	81.7	7.8	0.6
D[al]P	y = 148,885x + 66,921.6	0.9999	2–50	0.30	0.60	80.9	78.8	75.1	78.3	8.0	0.7
B[ghi]P	y = 135,674x + 42,856.7	0.9996	1–50	0.15	0.30	88.0	86.9	82.6	85.8	7.9	0.7
I[cd]P	y = 122,483x − 11,362.5	0.9997	1–50	0.28	0.56	83.2	81.1	79.9	81.4	7.7	0.6
D[ae]P	y = 240,462x + 24,541	0.9998	1–50	0.29	0.59	82.9	78.0	73.9	78.3	9.1	0.8
D[ai]P	y = 14,242x + 73,123.3	0.9995	1–50	0.13	0.25	82.0	80.4	73.3	78.6	9.2	0.8
D[ah]P	y = 127,819x + 43,472.9	0.9995	1–50	0.16	0.33	78.2	73.9	69.1	73.7	9.4	0.8

* Mean recovery, RSD, and HORRAT_R_ values of three different levels of sample fortification.

**Table 2:** Thermal efficiency of the smoking process of model homogenized smoked-pork sausages (%).

Thermal Efficiency for Particular Smoking Batches	Natural Casing	Cellulose Casing	Collagen Casing	Polyamide Casing	Control Sample
1	79.9	81.9	81.8	84.6	91.5
2	81.4	83.4	83.2	84.9	91.0
3	82.0	82.0	83.0	84.1	90.9
Mean thermal efficiency (% ± SD)	81.1 ± 1.1	82.5 ± 0.8	82.7 ± 0.7	84.5 ± 0.4	91.1 ± 0.4

**Table 3:** Mean content of PAHs in model smoked homogenized pork sausages of variant 1, including the type of casing and the part of the product (μg/kg ± SD).

PAH	Natural Casing		Cellulose Casing		Collagen Casing		Polyamide Casing	
	External Part	Internal Part	External Part	Internal Part	External Part	Internal Part	External Part	Internal Part
Phen ^x^	29.24 ± 2.49 ^A1^	8.93 ± 0.67 ^a1^	22.05 ± 2.04 ^B1^	4.85 ± 0.42 ^b1^	6.84 ± 0.55 ^C1^	0.72 ± 0.10 ^c1^	7.95 ± 0.76 ^C1^	0.81 ± 0.10 ^c1^
Anthr	4.99 ± 0.41 ^A2^	1.39 ± 0.08 ^a2^	4.02 ± 0.30 ^B2^	0.76 ± 0.07 ^b2^	0.99 ± 0.09 ^C2^	0.14 ± 0.02 ^c2^	1.07 ± 0.11 ^C2^	0.13 ± 0.03 ^c2^
F	14.85 ± 1.10 ^A3^	4.01 ± 0.22 ^a3^	11.18 ± 0.91 ^B3^	2.12 ± 0.14 ^b3^	2.70 ± 0.20 ^C3^	0.31 ± 0.05 ^c3^	2.85 ± 0.23 ^C3^	0.33 ± 0.06 ^c3^
Pyr	16.61 ± 1.23 ^A4^	4.43 ± 0.23 ^a4^	13.15 ± 1.12 ^B4^	2.33 ± 0.16 ^b4^	2.99 ± 0.22 ^C4^	0.33 ± 0.07 ^c4^	3.12 ± 0.30 ^C4^	0.36 ± 0.08 ^c4^
C[cd]P	nd ^w^	nd	nd	nd	nd	nd	nd	nd
B[a]A	2.84 ± 0.25 ^A5^	nd	2.15 ± 0.12 ^B5^	nd	nd	nd	nd	nd
Chr	2.67 ± 0.28 ^A6^	nd	1.78 ± 0.10 ^B6^	nd	nd	nd	nd	nd
5-MChr	nd	nd	nd	nd	nd	nd	nd	nd
B[j]F	nd	nd	nd	nd	nd	nd	nd	nd
B[b]F	2.25 ± 0.12 ^A7^	nd	1.67 ± 0.11 ^B7^	nd	nd	nd	nd	nd
B[k]F	2.41 ± 0.21 ^A8^	nd	1.88 ± 0.15 ^B8^	nd	nd	nd	nd	nd
B[a]P	2.34 ± 0.19 ^A9^	nd	1.79 ± 0.16 ^B9^	nd	nd	nd	nd	nd
D[al]P	nd	nd	nd	nd	nd	nd	nd	nd
D[ah]A	2.46 ± 0.18 ^A10^	nd	1.82 ± 0.20 ^B10^	nd	nd	nd	nd	nd
B[ghi]P	2.02 ± 0.22 ^A11^	nd	1.65 ± 0.18 ^B11^	nd	nd	nd	nd	nd
I[cd]P	nd	nd	nd	nd	nd	nd	nd	nd
D[ae]P	nd	nd	nd	nd	nd	nd	nd	nd
D[ai]P	nd	nd	nd	nd	nd	nd	nd	nd
D[ah]P	nd	nd	nd	nd	nd	nd	nd	nd
Σ 19 PAHs ^x^	82.68 ± 6.45 ^A12^	18.75 ± 1.19 ^a12^	63.14 ± 5.06 ^B12^	10.06 ± 0.78 ^b12^	13.52 ± 1.06 ^C12^	1.46 ± 0.23 ^c12^	14.99 ± 1.38 ^C12^	1.63 ± 0.26 ^c12^
Σ 15 heavy PAHs ^x^	16.99 ± 1.23 ^A13^	nd	12.74 ± 0.69 ^B13^	nd	nd	nd	nd	nd
Σ 4 light PAHs ^y^	65.69 ± 5.22 ^A14^	18.75 ± 1.19 ^a14^	50.40 ± 4.37 ^B14^	10.06 ± 0.78 ^b14^	13.52 ± 1.06 ^C14^	1.46 ± 0.23 ^c14^	14.99 ± 1.38 ^C14^	1.63 ± 0.26 ^c14^
Σ 4 marker-heavy PAHs ^z^	10.10 ± 0.68 ^A15^	nd	7.39 ± 0.39 ^B15^	nd	nd	nd	nd	nd

*n* = 12 (12 samples of every kind of product were analyzed, including 3 production batches). ^w^ nd—not detected. ^x^ Different capital or lowercase letters by the same number (e.g., A12, B12, C12 or a12, b12, c12) or a particular capital letter and its lowercase equivalent by the same number (e.g., A12 and a12) meaning one analyzed comparison, below the mean values of PAHs, indicate statistically significant differences between means at α = 0.05 level. ^y^ 4 light PAHs: Phen, Anthr, F, Pyr. ^z^ 4 marker-heavy PAHs: B[a]A, Chr, B[b]F, B[a]P.

**Table 4:** Mean content of PAHs in model smoked homogenized pork sausages in a natural casing, including the product variant and the part of the product (μg kg^−1^ ± SD).

	Natural Casing					
	Variant 1		Variant 2		Variant 3	
	External Part	Internal Part	External Part	Internal Part	External Part	Internal Part
Phen ^x^	29.24 ± 2.49 ^C16^	8.93 ± 0.67 ^b16^	36.74 ± 3.02 ^B16^	10.38 ± 0.85 ^ab16^	43.31 ± 3.35 ^A16^	11.95 ± 0.97 ^a16^
Anthr	4.99 ± 0.41 ^B17^	1.39 ± 0.08 ^c17^	5.84 ± 0.46 ^B17^	1.68 ± 0.09 ^b17^	6.95 ± 0.51 ^A17^	2.07 ± 0.16 ^a17^
F	14.85 ± 1.10 ^B18^	4.01 ± 0.22 ^c18^	16.68 ± 1.21 ^B18^	4.49 ± 0.28 ^b18^	19.45 ± 1.45 ^A18^	5.36 ± 0.31 ^a18^
Pyr	16.61 ± 1.23 ^C19^	4.43 ± 0.23 ^c19^	19.07 ± 1.35 ^B19^	5.12 ± 0.35 ^b19^	22.34 ± 1.59 ^A19^	6.19 ± 0.37 ^a19^
C[cd]P	nd ^w^	nd	nd	nd	nd	nd
B[a]A	2.84 ± 0.25 ^B20^	nd	3.35 ± 0.26 ^AB20^	nd	3.99 ± 0.31 ^A20^	0.18 ± 0.02 ^a20^
Chr	2.67 ± 0.28 ^B21^	nd	3.10 ± 0.27 ^B21^	nd	3.75 ± 0.30 ^A21^	0.16 ± 0.01 ^a21^
5-MChr	nd	nd	nd	nd	nd	nd
B[j]F	nd	nd	nd	nd	nd	nd
B[b]F	2.25 ± 0.12 ^C22^	nd	2.75 ± 0.17 ^B22^	nd	3.40 ± 0.22 ^A22^	0.20 ± 0.02 ^a22^
B[k]F	2.41 ± 0.21 ^C23^	nd	2.84 ± 0.22 ^B23^	nd	3.44 ± 0.26 ^A23^	0.19 ± 0.01 ^a23^
B[a]P	2.34 ± 0.19 ^C24^	nd	2.78 ± 0.20 ^B24^	nd	3.45 ± 0.24 ^A24^	0.24 ± 0.02 ^a24^
D[al]P	nd	nd	nd	nd	nd	nd
D[ah]A	2.46 ± 0.18 ^B25^	nd	2.85 ± 0.21 ^AB25^	nd	3.25 ± 0.27 ^A25^	0.25 ± 0.02 ^a25^
B[ghi]P	2.02 ± 0.22 ^B26^	nd	2.36 ± 0.20 ^B26^	nd	2.98 ± 0.23 ^A26^	0.23 ± 0.01 ^a26^
I[cd]P	nd	nd	nd	nd	nd	nd
D[ae]P	nd	nd	nd	nd	nd	nd
D[ai]P	nd	nd	nd	nd	nd	nd
D[ah]P	nd	nd	nd	nd	nd	nd
Σ 19 PAHs ^x^	82.68 ± 6.45 ^C27^	18.75 ± 1.19 ^c27^	98.37 ± 7.56 ^B27^	21.67 ± 1.57 ^b27^	116.32 ± 9.13 ^A27^	27.02 ± 1.92 ^a27^
Σ 15 heavy PAHs ^x^	16.99 ± 1.23 ^C28^	nd	20.04 ± 1.52 ^B28^	nd	24.27 ± 1.83 ^A28^	1.45 ± 0.11 ^a28^
Σ 4 light PAHs ^y^	65.69 ± 5.22 ^C29^	18.75 ± 1.19 ^c29^	78.33 ± 6.04 ^B29^	21.67 ± 1.57 ^b29^	92.05 ± 7.30 ^A29^	25.57 ± 1.81 ^a29^
Σ 4 marker-heavy PAHs ^z^	10.10 ± 0.68 ^C30^	nd	11.98 ± 0.90 ^B30^	nd	14.59 ± 1.07 ^A30^	0.78 ± 0.07 ^a30^

*n* = 12 (12 samples of every kind of product were analyzed, including 3 production batches). ^w^ nd—not detected. ^x^ Different capital or lowercase letters by the same number (e.g., A27, B27, C27 or a27, b27, c27) or a particular capital letter and its lowercase equivalent by the same number (e.g., A27 and a27) meaning one analyzed comparison, below the mean values of PAHs, indicate statistically significant differences between means at α = 0.05 level. ^y^ 4 light PAHs: Phen, Anthr, F, Pyr. ^z^ 4 marker-heavy PAHs: B[a]A, Chr, B[b]F, B[a]P.

**Table 5:** Mean content of PAHs in model smoked homogenized pork sausages in cellulose casing, including the product variant and the part of the product (μg kg^−1^ ± SD).

Pah	Cellulose Casing					
	Variant 1		Variant 2		Variant 3	
	External Part	Internal Part	External Part	Internal Part	External Part	Internal Part
Phen ^x^	22.05 ± 2.04 ^C31^	4.85 ± 0.42 ^b31^	26.75 ± 2.09 ^B31^	5.26 ± 0.45 ^b31^	32.42 ± 2.44 ^A31^	6.56 ± 0.50 ^a31^
Anthr	4.02 ± 0.30 ^B32^	0.76 ± 0.07 ^b32^	4.41 ± 0.33 ^AB32^	0.83 ± 0.09 ^ab32^	5.01 ± 0.38 ^A32^	0.95 ± 0.09 ^a32^
F	11.18 ± 0.91 ^C33^	2.12 ± 0.14 ^b33^	13.32 ± 0.96 ^B33^	2.32 ± 0.18 ^ab33^	14.89 ± 1.10 ^A33^	2.76 ± 0.21 ^a33^
Pyr	13.15 ± 1.12 ^C34^	2.33 ± 0.16 ^b34^	15.39 ± 1.23 ^B34^	2.56 ± 0.19 ^b34^	17.95 ± 1.35 ^A34^	3.14 ± 0.23 ^a34^
C[cd]P	nd ^w^	nd	nd	nd	nd	nd
B[a]A	2.15 ± 0.12 ^B35^	nd	2.48 ± 0.20 ^AB35^	nd	2.88 ± 0.24 ^A35^	nd
Chr	1.78 ± 0.10 ^C36^	nd	2.10 ± 0.15 ^B36^	nd	2.53 ± 0.17 ^A36^	nd
5-MChr	nd	nd	0.67 ± 0.07 ^B37^	nd	0.80 ± 0.10 ^B37^	nd
B[j]F	nd	nd	nd	nd	nd	nd
B[b]F	1.67 ± 0.11 ^C38^	nd	2.02 ± 0.16 ^B38^	nd	2.52 ± 0.18 ^A38^	nd
B[k]F	1.88 ± 0.15 ^B39^	nd	2.24 ± 0.18 ^AB39^	nd	2.65 ± 0.23 ^A39^	nd
B[a]P	1.79 ± 0.16 ^C40^	nd	2.15 ± 0.12 ^B40^	nd	2.53 ± 0.16 ^A40^	nd
D[al]P	nd	nd	0.34 ± 0.08 ^A41^	nd	0.41 ± 0.07 ^A41^	nd
D[ah]A	1.82 ± 0.20 ^B42^	nd	2.11 ± 0.15 ^AB42^	nd	2.46 ± 0.19 ^A42^	nd
B[ghi]P	1.65 ± 0.18 ^B43^	nd	1.88 ± 0.20 ^AB43^	nd	2.21 ± 0.22 ^A43^	nd
I[cd]P	nd	nd	nd	nd	nd	nd
D[ae]P	nd	nd	0.57 ± 0.10 ^A44^	nd	0.69 ± 0.11 ^A44^	nd
D[ai]P	nd	nd	nd	nd	nd	nd
D[ah]P	nd	nd	nd	nd	nd	nd
Σ 19 PAHs ^x^	63.14 ± 5.06 ^C45^	10.06 ± 0.78 ^b45^	76.43 ± 5.96 ^B45^	10.97 ± 0.90 ^b45^	89.95 ± 6.94 ^A45^	13.41 ± 1.01 ^a45^
Σ 15 heavy PAHs ^x^	12.74 ± 0.69 ^C46^	nd	16.56 ± 1.35 ^B46^	nd	19.68 ± 1.67 ^A46^	nd
Σ 4 light PAHs ^y^	50.40 ± 4.37 ^C47^	10.06 ± 0.78 ^b47^	59.87 ± 4.61 ^B47^	10.97 ± 0.90 ^b47^	70.27 ± 5.27 ^A47^	13.41 ± 1.01 ^a47^
Σ 4 marker-heavy PAHs ^z^	7.39 ± 0.39 ^C48^	nd	8.75 ± 0.63 ^B48^	nd	10.46 ± 0.75 ^A48^	nd

*n* = 12 (12 samples of every kind of product were analyzed, including 3 production batches). ^w^ nd—not detected. ^x^ Different capital or lowercase letters by the same number (e.g., A45, B45, C45 or a45, b45) or a particular capital letter and its lowercase equivalent by the same number (e.g., A45 and a45) meaning one analyzed comparison, below the mean values of PAHs, indicate statistically significant differences between means at α = 0.05 level. ^y^ 4 light PAHs: Phen, Anthr, F, Pyr. ^z^ 4 marker-heavy PAHs: B[a]A, Chr, B[b]F, B[a]P.

## Data Availability

The data presented in this study are available on request from the corresponding author.
